# The mutational spectrum of SARS-CoV-2 genomic and antigenomic RNA

**DOI:** 10.1098/rspb.2022.1747

**Published:** 2022-11-30

**Authors:** Lele Zhao, Matthew Hall, Mariateresa de Cesare, George MacIntyre-Cockett, Katrina Lythgoe, Christophe Fraser, David Bonsall, Tanya Golubchik, COVID-19 Genomics UK (COG-UK) Consortium, Luca Ferretti

**Affiliations:** ^1^ Big Data Institute, Li Ka Shing Centre for Health Information and Discovery, Nuffield Department of Medicine, University of Oxford, Oxford OX3 7LF, UK; ^2^ Human Technopole, Milan, Italy; ^3^ Wellcome Centre for Human Genetics, University of Oxford, Oxford OX3 7BN, UK; ^4^ Sydney Infectious Diseases Institute (Sydney ID), Faculty of Medicine and Health, University of Sydney, Sydney NSW 2006, Australia

**Keywords:** SARS-CoV-2, antigenomes, mutational spectrum, RNA editing

## Abstract

The raw material for viral evolution is provided by intra-host mutations occurring during replication, transcription or post-transcription. Replication and transcription of *Coronaviridae* proceed through the synthesis of negative-sense ‘antigenomes’ acting as templates for positive-sense genomic and subgenomic RNA. Hence, mutations in the genomes of SARS-CoV-2 and other coronaviruses can occur during (and after) the synthesis of either negative-sense or positive-sense RNA, with potentially distinct patterns and consequences. We explored for the first time the mutational spectrum of SARS-CoV-2 (sub)genomic and anti(sub)genomic RNA. We use a high-quality deep sequencing dataset produced using a quantitative strand-aware sequencing method, controlled for artefacts and sequencing errors, and scrutinized for accurate detection of within-host diversity. The nucleotide differences between negative- and positive-sense strand consensus vary between patients and do not show dependence on age or sex. Similarities and differences in mutational patterns between within-host minor variants on the two RNA strands suggested strand-specific mutations or editing by host deaminases and oxidative damage. We observe generally neutral and slight negative selection on the negative strand, contrasting with purifying selection in ORF1a, ORF1b and S genes of the positive strand of the genome.

## 1. Introduction

SARS-CoV-2 is the causative agent for COVID-19. Since the initial outbreak in Wuhan, China in December 2019, SARS-CoV-2 and many subsequent variants have caused a pandemic directly claiming the lives of 6.5 million people worldwide [1]. The tremendous efforts in combating the pandemic are unprecedented; vaccines and other therapeutics are developed and distributed with astonishing speed. However, as is characteristic of RNA viruses, SARS-CoV-2 has a high mutation rate and large population size within-host [2], giving it ample opportunity to escape immune responses [3] and pharmaceutical interventions and to adapt to the human host [4]. The pandemic is still ongoing, fuelled by new variants carrying problematic mutations that result in heightened transmissibility and immune escape [5,6]. Studying the mutational patterns and molecular biology of SARS-CoV-2 will enable us to better understand the observed evolutionary trajectories and their implications.

SARS-CoV-2 belongs to the *Coronaviridae* family, beta-coronavirus genus. The alpha- and beta-coronavirus genera also contain two well-known zoonotic viruses, severe acute respiratory syndrome coronavirus (SARS-CoV) and Middle East respiratory syndrome coronavirus, as well as four other common human coronaviruses (HCoV-OC43, HCoV-229E, HCoV-NL63 and HCoV-HKU1) [7]. Like other coronaviruses, SARS-CoV-2 is an enveloped, non-segmented, positive single-stranded RNA virus. It has a genome of approximately 30 kb in length, a size characteristic of several families within the virus order Nidovirales, but much larger than most of the other RNA viruses [8]. The coronaviruses are named for the many crown-resembling protein spikes on the outer capsid of the virion [9]. It is these spikes that bind to the cell receptor, angiotensin-converting enzyme 2, and initiate entry into a host cell [10,11]. After cleavage of the protein, the lipid membrane fuses with the cell membrane to release the nucleocapsid (N)-coated RNA genome into the cytoplasm of the host cell [11], where this positive strand of genomic RNA undergoes direct translation, replication and further transcription. The coronavirus genome encodes non-structural proteins (nsps), structural proteins and accessory proteins. The nsps are the products of proteolytic events of the polyproteins *pp1a* and *pp1ab*, translated from two open reading frames ORF1a and ORF1ab, respectively [12,13]. The production of *pp1a* and *pp1ab* from the same genomic sequence depends on a frameshift mechanism maintained by a slippery sequence [14]. A subset of the nsps come together to form the replication-transcription complex (RTC). The RTC synthesizes the complementary strand, full-length negative-strand RNA, to be used as template for genome replication. It also synthesizes subgenomic (sg) mRNAs, to code for the structural and accessory proteins. Specifically, the RTC uses discontinuous transcription to jump after encountering translational regulatory sequence (TRS-B) and stitch ORFs (excluding ORF1a and ORF1ab) with the 5′ end translational regulatory sequence (TRS-L) to produce negative-sense sgRNAs, which are then transcribed into positive sgmRNAs for protein translation [15].

**Figure 1.**
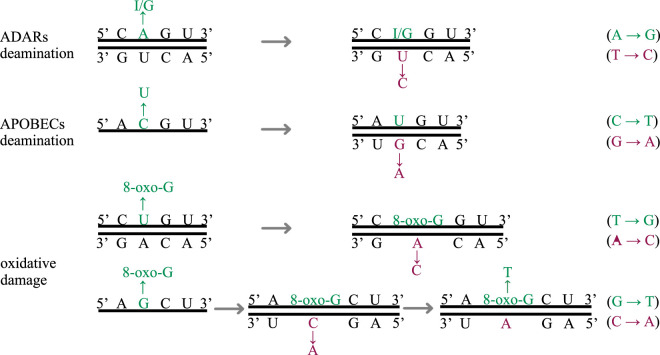
Example of the three types of mutations. The green-coloured bases show the original mutational changes; the magenta-coloured bases show the subsequent changes in base pairing. (Online version in colour.)

Two mammalian innate immune responses targeting external viral RNA through deaminase activities have been suggested to carry out genome editing of SARS-CoV-2 [16]. Adenosine deaminases acting on RNA (ADARs) change adenines into inosines (A to I) on double-stranded RNA [17], which would result in either an A > G mutation in the positive-strand products (full genome or sgmRNA), or a complementary U > C mutation if the first change took place on the negative template strand (figure 1). Apolipoprotein B mRNA-editing enzyme, catalytic polypeptide, or APOBEC deaminases mutate cytosines into uracils (C to U) on single-stranded RNA and single-stranded DNA [18]. Excessive C > U mutations (recorded in genome sequences as C > T by convention) that have been observed in the SARS-CoV-2 genome suggest high levels of RNA editing [16,19,20]. These are the direct observations of APOBEC deaminases acting on the positive strand, however, if APOBEC deaminases changed a C to U on the negative sgRNA of SARS-CoV-2, G > A mutations would be observed in the resulting complementary positive strands (figure 1). A third potential source of mutations is oxidative damage, where guanines in the nucleotide pool become 8-oxo-G [21,22], and preferentially pair with adenines during base pairing (figure 1).

Previous studies have focused on positive-sense sequences, generally represented by genomic RNA sequences, which dominate the conventional output from most next-generation RNA-sequencing protocols [16,19,23]. However, the intermediate negative-sense templates during within-host viral replication and transcription represent another important data source to describe the mutational patterns of this virus. For example, one can gather information on intra-cellular RNA editing patterns and also deduce the mutations that are purged or selected for by comparing the mutations occurring at corresponding genomic sites. With samples collected during the first few months of the UK's epidemic, and the quantitative strand-aware viral RNA deep sequencing method veSEQ [24], we obtained deep sequence information on both (sub)genomic and anti(sub)genomic strands of SARS-CoV-2. The antigenomic sequences consist of the full-length negative-sense genomes and subgenomic negative-sense RNAs. Despite naturally low amounts of antigenomic material in the samples, we are able to show that the variance in the number of strand consensus disagreements per sample is overdispersed, providing evidence of patient-specific responses to viral infections. We show strand-specific mutational spectra of SARS-CoV-2 and describe the types and levels of possible RNA editing taking place on both strands of the genome and compare their relative frequencies. We find generally neutral and weak negative selection on the antigenomic variants, contrasting with negative selection on the genomic variants of the ORF1a, ORF1b and S gene sequences.

## 2. Methods

### (a) Sample preparation and sequencing

Nasopharyngeal swabs were collected from symptomatic individuals upon admission to hospital and from healthcare workers during the period of March to June 2020. RNA extractions were performed in Oxford University Hospital or Basingstoke and North Hampshire Hospital as previously described [25]. Libraries were prepared following the veSEQ protocol [24] with modifications and sequenced on the Illumina Miseq and NovaSeq 6000 platforms (Illumina, CA, USA) at the Oxford Genomics Centre. Briefly, the viral RNA was extracted with QIAsymphony DSP Virus/Pathogen Kit (QIAGEN), Maxwell RSC Viral total nucleic acid kit (Promega), Reliaprep blood gDNA miniprep system (Promega) or Prepito NA body fluid kit (PerkinElmer). Two different extraction controls were present during the extraction, and neither affected sequencing. Libraries to be sequenced on the Illumina platforms were prepared with residual RNA samples after clinical testing, using the SMARTer Stranded Total RNA-Seq Kit v2-Pico Input Mammalian (Takara Bio USA, CA, USA) without RNA fragmentation. One crucial step of the SMARTer protocol is that different index sequences are appended to the two ends of the original template, thus retaining information of the strand orientation. The libraries were size-selected to retain fragments of at least 400 nt and underwent the targeted bait-capture process of the veSEQ protocol [24] to enrich and amplify SARS-CoV-2 sequences. The samples were sequenced to produce 150 bp or 250 bp paired-end reads. (For more specific details on the modified veSEQ protocol for SARS-CoV-2 and other measures to reduce sequence artefacts and increase data reproducibility, please see: [25])

### (b) Bioinformatic processing and analyses

After obtaining the raw sequencing outputs, the reads from bacterial and human sources were identified with Kraken v2 [26] using a custom database, and removed with filter_keep_reads.py from the Castanet (https://github.com/tgolubch/castanet) workflow [27]. The remaining viral and unclassified reads were quality-trimmed (Trimmomatic v0.36 [28]) and mapped to the SARS-CoV-2 reference genome (Wuhan-Hu-1, GenBank: NC_045512.2) using *shiver* v1.5.7 [29] with smalt as the mapper [30]. Because of a template-switching step in the SMARTer Stranded Total RNA-Seq Kit v2-Pico Input Mammalian (Takara Bio USA, CA, USA) protocol and its strand-aware design, each binary alignment (BAM) file was divided into two files: one containing read pairs mapped to the genomic strand (identified using SAM flags 83 or 163) and the other to the antigenomic strand (identified using SAM flags 99 and 147). Minor allele frequencies (MAFs) of each BAM file were summarized for all genomic positions using *shiver* [29] (tools/AnalysePileup.py).

To ensure high confidence in the analyses of genomic mutational spectra and reduce the stochasticity brought by variants in low viral load samples, we only examine the samples that produced 50 000 or more uniquely mapped reads. These correspond to high viral load samples, since the veSEQ protocol has been shown to retain a positive correlation between viral load and uniquely mapped reads. Upon further investigation, we subset the sequencing batches into two sets, batches 2–14 and batches 15–27. From our investigation, potential oxidative damage-related mechanisms induced a background of mutations (A > C and T > G) that dominated the negative-sense mutational spectrum of the later batches 15–27 at around 50% of all observed mutations ubiquitously (electronic supplementary material, figure S1). This was not problematic for the positive-sense mutational spectrum across all batches because the abundance of positive-sense genome copies is much higher, the MAF cutoff was 3%, and the oxidative damage-induced mutations are prevalent at lower frequencies (electronic supplementary material, figure S2); however, background oxidative damage effect is particularly prominent for the much less abundant negative-sense templates. Therefore, we only use the first 2–14 batches for our analyses and comparison of the two strands' mutational spectra. This brings our total number of high viral load samples with both positive- and negative-sense (sub)genomic coverage to 250.

All SARS-CoV-2 genomic sites (positions 1–29903) were examined. As suggested by our previous work, sites prone to generating low-frequency within-host variation *in vitro* [25] and variant sites shared by more than 20 samples were masked in within-host variation analysis. Positive-sense variant calling requires a minimum read depth of 100, and minimum MAF of 3%. Due to the naturally low abundance of negative-sense RNA in the samples, we required a minimum depth of 5 reads for negative-sense base (consensus and variant) calling (electronic supplementary material, figure S3). We determined the consensus at each site as the base with the highest read count and the minor variant at polymorphic sites as that with the second highest count. We defined cases where the two strands have the same consensus (variant) base at a site as an agreement between the strand consensuses (variants), and the alternative as a disagreement. Collectively in all 250 samples, the median per site negative-sense antigenomic read depth was 5.88 × 10^3^ reads [range 16–2.15 × 10^5^ reads], with approximately 3.46 × 10^2^–8.51 × 10^4^ reads recovered per sample. The median per site positive-sense genome read depth was 4.43 × 10^6^ reads [range 5.55 × 10^3^–3.85 × 10^7^ reads], with approximately 1.03 × 10^5^–1.46 × 10^7^ reads per sample.

The dependence between the number of disagreement sites between negative-sense and positive-sense consensus was modelled through a quasi-Poisson regression. We included epidemiological features such as age, sex, antigenomic read depth (in logscale) and number of unique mapped reads (also in logscale) as surrogate of viral load, enforcing also a linear dependence on sample antigenomic coverage in the model. The model has the form

$$\begin{array}{rl} & \log\left(\frac{\mathrm{number}\_\_\mathrm{of}\_\_\mathrm{disagreements}}{\mathrm{antigenomic}\_\_\mathrm{coverage}}\right)∼\mathrm{age}+\mathrm{sex}\\ & \;+\log(\mathrm{antigenomic}\_\_\mathrm{read}\_\_\mathrm{depth})\\ & \;+\log(\mathrm{number}\_\_\mathrm{of}\_\_\mathrm{unique}\_\_\mathrm{mapped}\_\_\mathrm{reads}). \end{array}$$

To disentangle the strong collinearity of the last two predictors, we performed a causal mediation analysis, considering the logscale antigenomic read depth as possible mediator of the effect of the logscale viral load, and modelling their dependence through a classical linear regression including age and sex:

$$\begin{array}{c} \log(\mathrm{antigenomic}\_\_\mathrm{read}\_\_\mathrm{depth})∼\mathrm{age}+\mathrm{sex}\;\\ +\log(\mathrm{number}\_\_\mathrm{of}\_\_\mathrm{unique}\_\_\mathrm{mapped}\_\_\mathrm{reads}).\; \end{array}$$

The dN/dS ratio for each coding region was calculated as

$$\frac{N_{s}^{c}/S_{s}^{c}}{N_{ref}^{c}/S_{ref}^{c}},$$

where $N_{s}^{c}\;$ is the total number of non-synonymous mutations from all samples within coding region *c*, $S_{s}^{c}\;$ is the total number of synonymous mutations from all samples within coding region *c*, $N_{\mathrm{ref}}^{c}\;$ is the total number of all possible non-synonymous mutations in SARS-CoV-2 reference genome [Wuhan-Hu-1, GenBank: NC_045512.2] within coding region *c* and $S_{\mathrm{ref}}^{c}\;$ is the total number of all possible synonymous mutations in SARS-CoV-2 reference genome [Wuhan-Hu-1, GenBank: NC_045512.2] within coding region *c*.

## 3. Results

### (a) Strand consensus disagreement

We first summarized the disagreements between the consensus bases on each strand within each sample. The median number of disagreement sites was 2 [range 0–16]. When fitted to a quasi-Poisson model to assess overdispersion weighted by antigenomic coverage we obtain a dispersion parameter of 2.7 with respect to the Poisson variance after controlling for age, sex and viral load. This suggests heterogeneity in mutations among patients possibly due to RNA editing [16,31], in which different hosts respond differently to viral infection. The significant dependency was on viral load (unique mapped reads: *p*-value = 4 × 10^−8^) and antigenomic read depth (*p*-value = 0.02), although a causal mediation model suggests that this dependency may be due to a mixture of direct effect of viral load and indirect effect through antigenomic read depth (*p*-value = 0.02), which is highly correlated to viral load (correlation coefficient = 0.65).

### (b) Strand-specific mutational patterns

To avoid interpreting sequencing artefacts as results, we need to identify mutation categories that contain high proportions of mutations likely to be produced by the sequencing protocol bias and *in silico* amplification error. We ran a sensitivity analysis by varying the depth support for variants on the negative-sense strand and compared the mutation spectrum between different depth supports (electronic supplementary material, figure S2). All consensus and variant pair mutation frequencies did not show strong variations when the depth support was increased, except for neg:A > T and neg:G > T. This suggests that these two mutational changes might be affected by systematic background bias, probably introduced by amplification or RT-PCR steps. Next, to further confirm the non-random nature of the negative-strand mutational spectrum, we generated the low-frequency mutational spectra of the positive strand—likely to be dominated by random sequencing errors—at variant thresholds: 0.005, 0.01 and 0.02 for comparison (electronic supplementary material, figure S2). In this comparison, neg:A > C and neg:T > A showed similar frequencies to the low-frequency random mutations on the positive strand, which means their frequencies are indistinguishable from random errors. Note that low-frequency mutations on the positive strand are dominated by T > G, while this is not the case for antigenomic mutations, supporting that most of the latter did not originate from sequencing errors or artefacts.

In addition to single-nucleotide site changes, we looked into dinucleotide mutational patterns of these single site changes, which presented a complex picture. On positive-strand genomic RNA, common mutations (MAF > = 3%) tend to be represented by a skewed set of dinucleotide changes and show markedly different patterns from low-frequency mutations (electronic supplementary material, figure S4). Dinucleotide changes on negative-strand antigenomic RNA are mostly equally distributed among dinucleotide pairs and show some similarities to the patterns for low-frequency positive-sense ones, but exhibit different proportions in some pairs, most prominently in dinucleotides that start with adenine or guanine. As expected, patterns for non-synonymous and synonymous dinucleotide changes are different as well (electronic supplementary material, figure S5). This suggests that such differences in mutational patterns are not artefactual but originate from different mutational mechanisms or differences in intra-host selection.

Next, we compare and speculate on the patterns between the *complemented* negative-strand and positive-strand mutational spectra for the non-artefactual single-nucleotide mutational categories. These patterns can be divided into three groups (electronic supplementary material, figure S6): (i) negative-strand mutation frequency is different from the positive-strand's frequency of random errors and is more similar to positive-strand non-random frequency. These pairs are A > G, C > T, G > A, G > C, T > C. (ii) Negative-strand mutation frequency is different from the positive-strands' random frequency and is also different from positive-strand non-random frequency. These mutations are A > C, C > G. (iii) Negative-strand (complemented) G > T mutation frequency is similar to the positive G > T random frequency, but is lower than the non-random positive frequency.

**Figure 2.**
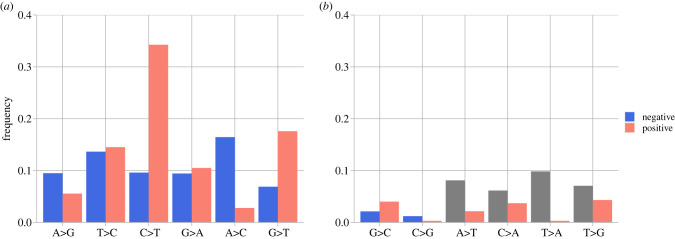
Consensus > variant pair frequencies for SARS-CoV-2 positive and complemented negative strands; (*a*) shows mutational changes attributable to known mechanisms (i.e. ADARs, APOBECs, oxidative damage); (*b*) shows other mutational changes, with A > T, C > A, T > A, T > G frequencies (grey) on the negative strand not interpreted. (Online version in colour.)

ADARs recognize and deaminase double-stranded RNA, and when directly sequenced, the inosines from ADARs are recognized as guanines, as inosines preferably pair with cytosines [32]. Therefore, a direct excess of A > G pairs on the strands and an excess of T > C in the complementary strand would indicate ADARs activities. We observe high levels of T > C changes, but the signal is less so for A > G (figure 2). APOBECs tend to induce the change C > T on single-stranded RNA and DNA. When C > T changes take place on the complementary strand, we would observe G > A after base pairing. We see matching relatively high G > A frequencies on both strands. However, we also see an excess of positive-strand C > T which is not mirrored by the negative strand, perhaps indicating high levels of post-transcriptional changes (figure 2). There are also excess A > C (neg: T > G) mutations arising on the negative sense but being purged on the positive strand. While T > G mutations are often attributed to oxidative damage, we do not see the complementary changes being retained in the positive sense. More G > T mutations are also observed for the positive strands without matching frequencies on the negative strand. Both A > C (neg:T > G) and G > T are possibly caused by reactive oxygen species damage to guanines in the nucleotide pool. The product 8-oxo-G pairs with adenines, thus resulting in T > G and G > T in different scenarios (figure 1). Other mutational changes are not discussed here because of low-frequency and artefact-related uncertainties.

**Figure 3.**
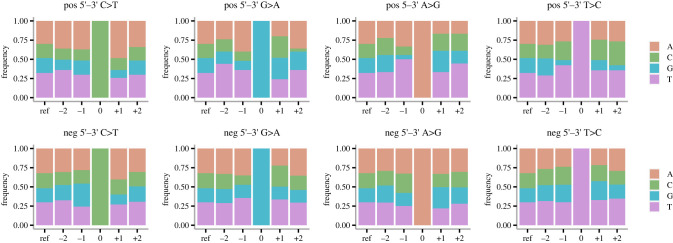
Genomic context of candidate sites for RNA editing on the positive (top row) and non-complemented negative (bottom row) strands. The site of change is labelled 0, with the base positions to the 5′ direction labelled −2 and −1, base positions to the 3′ direction labelled + 1 and + 2. The ‘ref’ columns show the genomic content of the SARS-CoV-2 reference sequence [GenBank ID: NC_045512.2]. (Online version in colour.)

Among all 250 samples, we called 280 genomic variable sites (MAF > = 3% and minimum coverage 100 reads) and 7196 antigenomic variable sites (minimum 5 reads to call a variant base for the negative-sense strand). For the positive-strand consensus > variant changes, there is an abundance of A and T up- and downstream of a C > T change (figure 3), which is the expected sequence context for APOBEC1 deaminases [16,33,34]. There is also a drastic depletion of base G at position −1 of positive-strand A > G change (figure 3), which is the common ADARs-induced pattern observed in human transcripts [16]. However, the negative-strand C > T and A > G changes do not show the same typical genomic context indicative of deamination, and this pattern is consistent for negative-strand variant sites with increased support (electronic supplementary material, figure S7). With minimum 10, 20 and 50 reads to call a variant base on the negative strand, there are 3276, 1273 and 277 variable sites remaining respectively. The sequence context surrounding negative-strand C > T and A > G changes do not show the expected APOBEC and ADARs context. This could be partly due to the detection limit imposed by the low abundance of the negative-sense templates.

### (c) Strand-specific selection patterns

**Figure 4.**
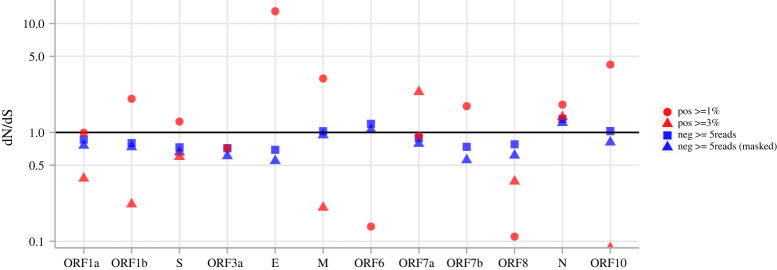
dN/dS of SARS-CoV-2 coding regions computed with differently filtered variants. Red circles are from positive-strand variant sites with no less than 1% MAF; red triangles are from positive-strand variant sites with no less than 3% MAF. Blue squares are from negative-strand variant sites with at least five reads supporting the minor variant, blue triangles are the same sites but with A > T, C > A, T > A, T > G sites masked. ‘Pos > = 3%’ dN/dS = Inf are not shown for ORF3a, ORF6 and ORF7b. No ‘Pos > = 3%’ variant sites were called within E; therefore, dN/dS is not shown. (Online version in colour.)

To check if the discrepancy in the strand-specific mutational patterns is associated with strand-specific selective pressure, we calculated dN/dS for each coding region (figure 4; electronic supplementary material, table S1). While dN/dS is difficult to interpret for within-host mutations, it is still indicative of selective processes at the protein level. The dN/dS ratios across the genome in the negative sense detected from different depth support suggest consistent neutral to weak negative selection (dN/dS < 1 and approx. equal to 1), except for the ORF6 and N genes being slightly higher than 1. While the high confidence minor variants (MAF > = 3%) on the positive strand show strong purifying selection on the ORF1a, ORF1b, S gene as different from the dN/dS ratios from variants above 1% on the positive strand. It is interesting to note that the N gene on the positive strand also displayed dN/dS above 1, indicating the presence of positive selection. Other coding regions contained low numbers of high confidence variants above 3%, therefore comparisons cannot be made reliably. These results suggest generally neutral and weak negative selection on the negative strand of SARS-CoV-2 and purifying selection on ORF1a, ORF1b and S genes of the positive-strand genome.

**Figure 5.**
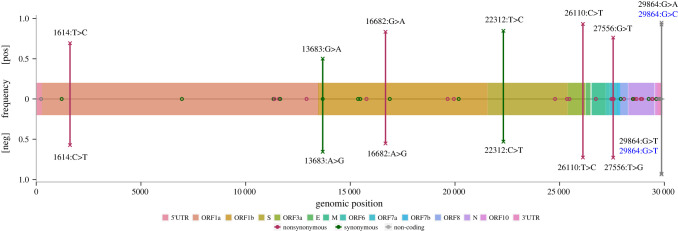
SARS-CoV-2 polymorphic sites shared by both strands. The *x*-axis indicates the genomic positions of the sites, the *y*-axis ([pos] for positive strand, [neg] for negative strand) indicates the consensus base frequencies of the mismatching sites. 'O's mark sites with complementing consensus-variant pairs from the two strands (base frequencies not shown). 'X's mark consensus-variant pair mismatching sites, with positive consensus frequency on the top, negative consensus frequency on the bottom, connected by a vertical line. Each mismatching site is labelled with genomic position and positive consensus > variant in the top half of the plot and complemented negative consensus > variant (in the positive sense) in the lower half of the plot. (Online version in colour.)

To further explore the difference in mutational patterns, we focus on 52 sites from 41 different samples that are found to be polymorphic on both strands (figure 5; electronic supplementary material, figure S8). Forty-four out of the 52 (85%) sites have complementing consensus and minor variants from the two strands, meaning that when the consensus-variant pair is C > T on the positive strand, the complemented pair on the negative strand is also C > T. Of the matching pairs, 7 are from ORF1a, 8 from ORF1b, 2 from S, 3 from ORF7a, 2 from ORF8, 15 from N, 3 from the 3′ UTR region, and 1 each from 5′UTR, ORF3a and M and ORF10. There is one mismatching pair located in ORF1a, S, ORF3a, ORF7a, two pairs in ORF1b and 3′UTR, respectively. Computations of dN/dS across these polymorphic sites show evidence of purifying selection in ORF1a, ORF1b and S genes (dN/dS = 0.26), and positive selection on the rest of the genome (dN/dS = 1.47).

Focusing on the base frequencies at the mismatching positions, the negative consensus bases are mostly between 53.0% and 72.9% in frequency (except for position 29 864 in the 3′UTR at 93.7% and 92.8%), while the positive consensus bases are between 50.1% and 94.8% in frequency. The consensus-variant pairs are the same two bases (except for position 29 864) for these sites but had altered which one was the majority on the two strands (figure 5). This suggests that the base frequencies on the two strands are probably fluctuating or converging within-host, as the difference is probably caused by stochasticity from the negative strand. The distribution of these polymorphic sites appears to be heterogeneous across the genome, with a higher density towards the last third of the genome (electronic supplementary material, figure S8). This is likely to be due to the intertwined effects of the relative abundance of different lengths of mRNA in the intra-cellular pool and the number of sequencing reads. The absolute depth of sequencing (electronic supplementary material, figure S3) and depth ratio of antigenome against genome (electronic supplementary material, figure S9) is low for the first two-thirds of the genome. This is consistent with the difference in abundance of full-length RNA and subgenomic mRNAs of the structural and accessory genes, the latter of which make up the last one-third of the genome. Because discontinuous transcription jumps at TRS-B, and ORF1a/ORF1ab is a long ORF without a TRS-B in the middle, ORF1a/ORF1ab only exist in full-length positive-sense RNAs; while there is a TRS-B at the start of every structural and accessory gene [35]. Therefore, the number of mutations detected in the first two-thirds of the genome is intrinsically lower than that of the last one-third.

## 4. Discussion

We present the mutational patterns of both the positive and negative strands of the SARS-CoV-2 genome. From RNA-seq reads, we checked the consensus agreement of positive and negative strands for 250 samples from acute infection with high viral load. Our analysis shows different patient-specific responses to viral replication. Next, we show evidence of both APOBEC C > T (G > A) and ADARs A > G (T > C) deamination activities on the positive- and negative-sense RNAs, with strong genomic context supporting the changes on the positive strand but not on the negative strand. We also show mutational changes caused by oxidative damage (i.e. A > C, G > T) on the two strands. Lastly, by computing the dN/dS ratio for all mutational changes, we observe overall neutral and weak purifying selection on the negative-sense antigenome, and overall purifying selection in ORF1a, ORF1b and S regions of the positive-sense genome. For those genomic sites that are polymorphic on both strands, we see a consistent match of the mutational spectrum of the two strands, with purifying selection on ORF1a, ORF1b and S genes of the positive-strand genome.

Our results suggest varying host responses to infection, given the variability in the strand consensus disagreement among samples that does not depend on age or sex. This indicates that patients may exhibit different levels of strand-specific mutations or RNA editing on the virus anti(sub)genome. Different sites of viral replication or sites of infection may result in different levels of host response, e.g. tissue-specific deaminase activities [31]. An infection of a type of tissue with high deaminase activities will lead to high frequencies of occurrences of mutation in the viral sample analysed. Mechschryakova and colleagues showed the heightened expression of APOBEC4 in cell types, such as respiratory tract epithelium cells, likely frequently targeted by SARS-CoV-2.

As viruses regularly infecting tissues with high APOBEC activity will carry signatures of previous infections in their genomes [36], many studies have summarized the mutational spectrum of the SARS-CoV-2 genome and found evidence of RNA editing. We have provided further evidence of it on the positive-strand mutational spectrum, while the picture from the matching negative-strand mutational spectrum is more ambiguous. The high frequencies of C > U (G > A) mutations suggest APOBEC-mediated RNA editing, as has been pointed out on numerous accounts of SARS-CoV-2 and other coronaviruses. However, while some [16], as well as our study, show evidence of A to I/G (U to C) mutations characteristic of ADARs present in SARS-CoV-2 infection, many other studies searched but found limited [37], or even no ADARs-mediated activities [35,38]. On the other hand, the excess of some mutations, such as A > G, on the negative strand compared to the positive one does not look consistent with patterns of ADARs editing and may have a different mutational origin. In addition to the deamination, we also see a high frequency of negative-strand T > G, which is A > C in the positive sense. This can be caused by the oxidative damage of guanines in the nucleotide pool producing 8-oxo-guanines, which preferentially pair with adenines, and then downstream replaces the original Adenine-Thymine pair with a Cytosine-Guanine pair [22]. Another mutation also possibly caused by reactive oxygen species is G > T (C > A), which we observe to be in high frequency on the positive strand, and has been reported in other studies as well [39,40]. In addition to the discussed deamination and potential oxidative damage-induced single-nucleotide site changes, there could be other explanations for the mutational spectra we observe. Examining dinucleotide biases may provide a different range of genomic context for the polymorphic sites. Dinucleotide mutational spectra are complex but show different patterns for genomic and antigenomic reads, as well as for synonymous and non-synonymous ones, suggesting that different combinations of mechanisms are involved in the origin or selection of genomic, antigenomic and subgenomic mutations.

The strand-aware library preparation methods used for the samples in our data provided the opportunity to identify reads from both the positive- and negative-sense (sub)genomes of SARS-CoV-2. Sequences from the negative strand can provide information from the previously unobserved step and shed more light on host-responsive RNA editing patterns and the mutations potentially purged within-hosts due to evolutionary pressures. Although the strand-aware library preparation has not been systematically validated for SARS-CoV-2 in terms of its recovery of the negative strand and we have seen a background noise of the positive sense reads mapping in the opposite direction for HIV samples, the same patterns of noise were not observed for SARS-CoV-2 samples. We compared the mutational patterns from the two strands, which are known to serve different purposes in the virus replication cycle and are of very different levels of abundance within the host cytoplasm [41]. Our positive to negative genome mapping ratio is between 100 and 1000, which can be compared to Sawicki *et al*. [41] stating a 50–100 fold difference in abundance of the two strands. Our protocol also cannot separate positive-sense genomic RNA from positive-sense subgenomic RNA fragments. This brings additional complexity to the interpretation of our findings. Variability among genomic sequences can be interpreted either as polymorphisms in the viral population (in which case it should also appear in antigenomic sequences if coverage would be high enough) or as mRNA sequence diversity generated within hosts by the transcription process.

RNA viruses like SARS-CoV-2 are characterized by high mutation rates, occurring both on genomic and antigenomic RNA. Within-host RNA editing acts as a further source of mutation in addition to polymerase-induced mutations and may have a long-lasting impact on the evolution of viral pathogens, as have seen with the depletion of cytosine as well as APOBEC acting motifs in other human circulating coronaviruses [42,43]. To better understand within-host selection, it is useful to incorporate the mutational spectrum of the negative sense of coronaviruses into future evolutionary analysis. Because of the low abundance within-host, negative-strand replication-transcription templates of positive single-stranded RNA viruses are often missed in sequencing, and targeted capture and amplification of these strands of RNA may provide a detailed and valuable piece to understanding the mutational spectra during the life cycle of these viruses.

## Data Availability

We used only publicly available data. The dataset analysed in this paper has been published in [25]. Specifically, the BAM files are available as part of European Nucleotide Archive (ENA) study PRJEB37886. The supplementary figures and table are provided in the electronic supplementary material [44].

## References

[1] World Health Organization. 2022 COVID-19 weekly epidemiological update, edition 110, 21 September. https://apps.who.int/iris/bitstream/handle/10665/363125/nCoV-weekly-sitrep21Sep22-eng.pdf.

[2] Stern A, Andino R. 2016 Viral evolution: it is all about mutations. In Viral pathogenesis: from basics to systems biology (eds MG Katze, MJ Korth, GL Law, N Nathanson), pp. 233-240. New York, NY: Academic Press.

[3] Eguia RT, Crawford KHD, Stevens-Ayers T, Kelnhofer-Millevolte L, Greninger AL, Englund JA, Boeckh MJ, Bloom JD. 2021 A human coronavirus evolves antigenically to escape antibody immunity. PLoS Pathog. **17**, e1009453. (10.1371/journal.ppat.1009453) 33831132 PMC8031418

[4] Bordería AV, Stapleford KA, Vignuzzi M. 2011 RNA virus population diversity: implications for inter-species transmission. Curr. Opin. Virol. **1**, 643-648. (10.1016/j.coviro.2011.09.012) 22440922

[5] Davies NG et al. 2021 Estimated transmissibility and impact of SARS-CoV-2 lineage B.1.1.7 in England. Science **372**, eabg3055. (10.1126/science.abg3055) 33658326 PMC8128288

[6] Liu Y, Rocklöv J. 2021 The reproductive number of the delta variant of SARS-CoV-2 is far higher compared to the ancestral SARS-CoV-2 virus. J. Travel Med. **28**, taab124. (10.1093/jtm/taab124) 34369565 PMC8436367

[7] Paules CI, Marston HD, Fauci AS. 2020 Coronavirus infections-more than just the common cold. J. Am. Med. Assoc. **323**, 707-708. (10.1001/jama.2020.0757) 31971553

[8] Gorbalenya AE, Enjuanes L, Ziebuhr J, Snijder EJ. 2006 Nidovirales: evolving the largest RNA virus genome. Virus Res. **117**, 17-37. (10.1016/j.virusres.2006.01.017) 16503362 PMC7114179

[9] TYRELL, and DA. 1968 Coronaviruses. Nature **220**, 650.

[10] Zhou P et al. 2020 A pneumonia outbreak associated with a new coronavirus of probable bat origin. Nature **579**, 270-273. (10.1038/s41586-020-2012-7) 32015507 PMC7095418

[11] Hoffmann M et al. 2020 SARS-CoV-2 cell entry depends on ACE2 and TMPRSS2 and is blocked by a clinically proven protease inhibitor. Cell **181**, 271-80.e8. (10.1016/j.cell.2020.02.052) 32142651 PMC7102627

[12] Brian DA, Baric RS. 2005 Coronavirus genome structure and replication. Curr. Top. Microbiol. Immunol. **287**, 1-30. 15609507 10.1007/3-540-26765-4_1 PMC7120446

[13] Masters PS. 2006 The molecular biology of coronaviruses. Adv. Virus Res. **66**, 193-292. (10.1016/S0065-3527(06)66005-3) 16877062 PMC7112330

[14] V'kovski P, Kratzel A, Steiner S, Stalder H, Thiel V. 2021 Coronavirus biology and replication: implications for SARS-CoV-2. Nat. Rev. **19**, 155-170. (10.1038/s41579-020-00468-6) PMC7592455 33116300

[15] Sola I, Almazán F, Zúñiga S, Enjuanes L. 2015 Continuous and discontinuous RNA synthesis in coronaviruses. Annu. Rev. Virol. **2**, 265-288. (10.1146/annurev-virology-100114-055218) 26958916 PMC6025776

[16] Di Giorgio S, Martignano F, Torcia MG, Mattiuz G, Conticello SG. 2020 Evidence for host-dependent RNA editing in the transcriptome of SARS-CoV-2. Sci. Adv. **6**, eabb5813. (10.1126/sciadv.abb5813) 32596474 PMC7299625

[17] Eisenberg E, Levanon EY. 2018 A-to-I RNA editing - immune protector and transcriptome diversifier. Nat. Rev. **19**, 473-490. (10.1038/s41576-018-0006-1) 29692414

[18] Harris RS, Dudley JP. 2015 APOBECs and virus restriction. Virology **479–480**, 131-145. (10.1016/j.virol.2015.03.012) PMC4424171 25818029

[19] Simmonds P. 2020 Rampant C→U hypermutation in the genomes of SARS-CoV-2 and other coronaviruses: causes and consequences for their short- and long-term evolutionary trajectories. mSphere **5**, e00408-20. (10.1128/mSphere.00408-20) 32581081 PMC7316492

[20] Matyášek R, Kovařík A. 2020 Mutation patterns of human SARS-CoV-2 and bat RaTG13 coronavirus genomes are strongly biased towards C>U transitions, indicating rapid evolution in their hosts. Genes **11**, 761. 32646049 10.3390/genes11070761 PMC7397057

[21] Kong Q, Lin CLG. 2010 Oxidative damage to RNA: mechanisms, consequences, and diseases. Cell. Mol. Life Sci. **67**, 1817-1829. (10.1007/s00018-010-0277-y) 20148281 PMC3010397

[22] Poetsch AR. 2020 The genomics of oxidative DNA damage, repair, and resulting mutagenesis. Comput. Struct. Biotechnol. J. **18**(January), 207-219. (10.1016/j.csbj.2019.12.013) 31993111 PMC6974700

[23] Tonkin-Hill G et al. 2021 Patterns of within-host genetic diversity in SARS-CoV-2. eLife **10**, e66857. (10.7554/eLife.66857) 34387545 PMC8363274

[24] Bonsall D et al. 2020 A comprehensive genomics solution for HIV surveillance and clinical monitoring in low-income settings. J. Clin. Microbiol. **58**, e00382-20. (10.1128/JCM.00382-20) 32669382 PMC7512176

[25] Lythgoe KA et al. 2021 SARS-CoV-2 within-host diversity and transmission. Science **372**, eabg0821. (10.1126/science.abg0821) 33688063 PMC8128293

[26] Wood DE, Lu J, Langmead B. 2019 Improved metagenomic analysis with Kraken 2. Genome Biol. **20**, 257. (10.1186/s13059-019-1891-0) 31779668 PMC6883579

[27] Goh C et al. 2019 Targeted metagenomic sequencing enhances the identification of pathogens associated with acute infection. *bioRxiv*. (10.1101/716902)

[28] Bolger AM, Lohse M, Usadel B. 2014 Trimmomatic: a flexible trimmer for illumina sequence data. Bioinformatics **30**, 2114-2120. (10.1093/bioinformatics/btu170) 24695404 PMC4103590

[29] Wymant C et al. 2018 Easy and accurate reconstruction of whole HIV genomes from short-read sequence data with shiver. Virus Evol. **4**, vey007. (10.1093/ve/vey007) 29876136 PMC5961307

[30] Sanger Institute. 2021 Tools directory. See https://www.sanger.ac.uk/science/tools.

[31] Meshcheryakova A, Pietschmann P, Zimmermann P, Rogozin IB, Mechtcheriakova D. 2021 AID and APOBECs as multifaceted intrinsic virus-restricting factors: emerging concepts in the light of COVID-19. Front. Immunol. **12**, 690416. (10.3389/fimmu.2021.690416) 34276680 PMC8282206

[32] Alseth I, Dalhus B, Bjørås M. 2014 Inosine in DNA and RNA. Curr. Opin Genet. Dev. **26**, 116-123. (10.1016/j.gde.2014.07.008) 25173738

[33] Rosenberg BR, Hamilton CE, Mwangi MM, Dewell S, Nina Papavasiliou F. 2011 Transcriptome-wide sequencing reveals numerous APOBEC1 mRNA editing targets in transcript 3′ UTRs. Nat. Struct. Mol. Biol. **18**, 230-236. (10.1038/nsmb.1975) 21258325 PMC3075553

[34] Anant S, Davidson NO. 2000 An AU-rich sequence element (UUUN[A/U]U) downstream of the edited C in Apolipoprotein B mRNA is a high-affinity binding site for Apobec-1: binding of Apobec-1 to this motif in the 3′ untranslated region of c-Myc increases mRNA stability. Mol. Cell. Biol. **20**, 1982-1992. (10.1128/MCB.20.6.1982-1992.2000) 10688645 PMC110815

[35] Kim D, Lee JY, Yang JS, Kim JW, Narry Kim V, Chang H. 2020 The architecture of SARS-CoV-2 transcriptome. Cell **181**, 914-921. (10.1016/j.cell.2020.04.011) 32330414 PMC7179501

[36] Wei Y, Silke JR, Aris P, Xia X. 2020 Coronavirus genomes carry the signatures of their habitats. PLoS ONE **15**, e0244025. (10.1371/journal.pone.0244025) 33351847 PMC7755226

[37] Li J et al. 2022 Two-step fitness selection for intra-host variations in SARS-CoV-2. Cell Rep. **38**, 110205. (10.1016/j.celrep.2021.110205) 34982968 PMC8674508

[38] Klimczak LJ, Randall TA, Saini N, Li JL, Gordenin DA. 2020 Similarity between mutation spectra in hypermutated genomes of rubella virus and in SARS-CoV-2 genomes accumulated during the COVID-19 pandemic. PLoS ONE **15**, e0237689. (10.1371/journal.pone.0237689) 33006981 PMC7531822

[39] Mourier T, Sadykov M, Carr MJ, Gonzalez G, Hall WW, Pain A. 2021 Host-directed editing of the SARS-CoV-2 genome. Biochem. Biophys. Res. Commun. **538**, 35-39. (10.1016/j.bbrc.2020.10.092) 33234239 PMC7643664

[40] Graudenzi A, Maspero D, Angaroni F, Piazza R, Ramazzotti D. 2021 Mutational signatures and heterogeneous host response revealed via large-scale characterization of SARS-CoV-2 genomic diversity. iScience **24**, 102116. (10.1016/j.isci.2021.102116) 33532709 PMC7842190

[41] Sawicki SG, Sawicki DL, Siddell SG. 2007 A contemporary view of coronavirus transcription. J. Virol. **81**, 20-29. (10.1128/JVI.01358-06) 16928755 PMC1797243

[42] Poulain F, Lejeune N, Willemart K, Gillet NA. 2020 Footprint of the host restriction factors APOBEC3 on the genome of human viruses. PLoS Pathog. **16**, e1008718. (10.1371/journal.ppat.1008718) 32797103 PMC7449416

[43] Ratcliff J, Simmonds P. 2021 Potential APOBEC-mediated RNA editing of the genomes of SARS-CoV-2 and other coronaviruses and its impact on their longer term evolution. Virology **556**, 62-72. (10.1016/j.virol.2020.12.018) 33545556 PMC7831814

[44] Zhao L et al. 2022 Supplementary material from: The mutational spectrum of SARS-CoV-2 genomic and antigenomic RNA. *Figshare*. (10.6084/m9.figshare.c.6266164) PMC9667359 36382519

